# A Fiber Bragg Grating Borehole Deformation Sensor for Stress Measurement in Coal Mine Rock

**DOI:** 10.3390/s20113267

**Published:** 2020-06-08

**Authors:** Wusheng Zhao, Kun Zhong, Weizhong Chen

**Affiliations:** 1 State Key Laboratory of Geomechanics and Geotechnical Engineering, Institute of Rock and Soil Mechanics, Chinese Academy of Sciences, Wuhan 430071, China; zhongkun17@mails.ucas.edu.cn (K.Z.); wzchen@whrsm.ac.cn (W.C.); 2 University of Chinese Academy of Science, Beijing 100049, China; 3 Postdoctoral Workstation, Yankuang Group Company Limited, Zoucheng 273500, China

**Keywords:** fiber Bragg grating, borehole deformation, built-in check, in situ stress, induced stress

## Abstract

A borehole deformation sensor for long-term stress monitoring in coal mine rock based on optical fiber Bragg gratings (FBGs) is presented. The sensor converts borehole deformation into optical fiber strain by using four rings. For each ring, two FBGs are bonded with the ring to measure the borehole deformation, and a reference FBG free from mechanical load is introduced to remove the temperature effect. Two simple checks on the test data can be performed to improve the test accuracy. Laboratory and field tests were conducted to validate the accuracy and long-term performance of the sensor. The results show that the sensor is capable of measuring stress in rock with good accuracy, and it performs well over a long period of time in coal mines. The developed sensor provides an approach for the long-term monitoring of stress changes in coal mine rock.

## 1. Introduction

Stress-induced instability is one of several major concerns for the safe construction and operation of coal mines. The measurement of in situ stresses is important in the design of coal mines, and monitoring of how stress changes with time is also vital when assessing the long-term performance of coal mines. Therefore, it is of significance to develop a sensor for both the determination of in situ stress and the long-term monitoring of stress changes in coal mine rock. 

Over the last few decades, a large number of studies on determining in situ stresses have been conducted. Various techniques [1,2,3,4,5] for stress determination have been proposed, and many measuring sensors [6,7,8,9] have been developed. The sensors can be divided into three groups: stiff or solid cells [10], borehole deformation gauges [7], and soft inclusion cells [6]. Because the borehole deformation gauge can be calibrated to ensure its accuracy, it is regarded as one of the most reliable and accurate sensors for stress determination. For these test techniques and sensors, Sjoberg et al. [11] introduced in detail the methods of data analysis and stress calculation. Han and Yin [12] proposed an intelligent method to determine in-situ stresses, and the method could solve the issue of the multiple solutions for a certain borehole deformation. However, most of the existing sensors are used for the determination of the in situ stress, and they do not allow the continuous observation of stress changes or do not perform well in the long-term monitoring of stress changes [13], especially in harsh environments like coal mines. For long-term monitoring of stress changes in coal mine rock, the sensor is affected by electromagnetic interference, drift, internal corrosion, and vibration in coal mines. For the existing sensors, the fundamental sensing elements are electrical strain gauges that are sensitive to the electromagnetic interference, corrosion, and vibration. Moreover, the electric sensors and cables may affect the safety of high-gas coal mines. The adverse conditions and safety requirements in coal mines make the existing sensors inadequate for the long-term monitoring of stress changes in mine rock. The fiber Bragg grating (FBG) sensors have several advantages compared to conventional electrical strain gauges: a small size, no electromagnetic interference, immunity to intensity vibrations and corrosion, and intrinsic safety. This inherent characteristic makes the FBG sensors very attractive for application in harsh environments [14], and long-term measurements using FBGs as sensing elements have been possible. FBG sensors are becoming more and more popular for geotechnical applications, and they are used to measure the strain [15,16], stress [17,18,19], displacement [20,21], and deformation [22,23,24] of the geotechnical media. In recent years, some unconventional FBG sensors, such as chirped FBG sensors [25] and tilted FBG sensors [26], are also proposed, and they show some advantages over traditional FBG sensors in some aspects.

The main objective of this study is to develop a FBG borehole deformation sensor for both the determination of in situ stresses and the long-term monitoring of stress changes in coal mine rock. First, the measurement principles and structure of the FBG borehole deformation sensor were introduced. Subsequently, the feasibility of the presented FBG sensor was validated through a series of tests. At last, the sensor was applied in a coal mine to measure the in situ stresses and monitor the stress changes in the roof. The presented FBG borehole deformation sensor can provide two built-in checks on the accuracy of the test, which can ensure the reliability and accuracy of test results. In addition, the FBG sensor can overcome the harsh environment in coal mines over a long period of time. Therefore, the FBG sensor may provide an approach for both the determination of the full in situ stress tensor and the long-term monitoring of stress changes in coal mines.

## 2. Measurement Principles of Stress in Mine Rock

### 2.1. Relationship Between Stresses in Rock and Borehole Deformations

**Figure 1:**
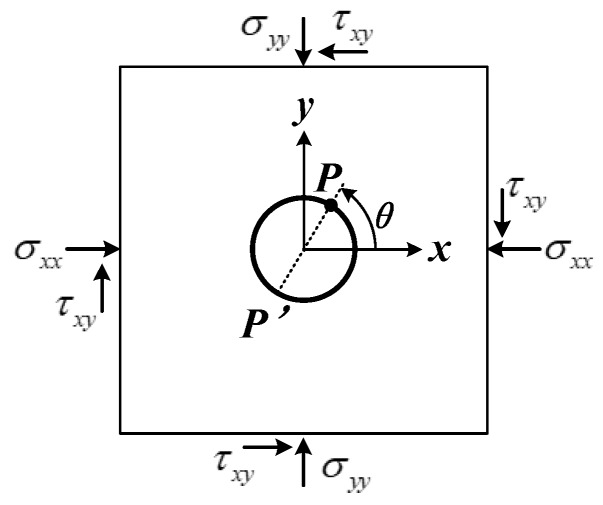
Borehole in a plane-strain condition.

As shown in Figure 1, the rock is assumed to be linearly elastic, homogeneous and continuous. A borehole is drilled in the rock, and the borehole is assumed to be in a plane strain (or stress) condition. According to the elasticity theory, the relationship between the stress components ($\sigma_{xx},\;\sigma_{yy},\;\tau_{xy})$ in the plane perpendicular to the borehole axis and the borehole deformations can be written as follows:

(1) $$\left[\begin{array}{l} \sigma_{xx}\\ \sigma_{yy}\\ \tau_{xy} \end{array}\right]={\left[\begin{array}{ccc} f_{1}^{1} & f_{2}^{1} & f_{3}^{1}\\ f_{1}^{2} & f_{2}^{2} & f_{3}^{2}\\ f_{1}^{3} & f_{2}^{3} & f_{3}^{3} \end{array}\right]}^{-1}\left[\begin{array}{l} U_{1}\\ U_{2}\\ U_{3} \end{array}\right]$$

where

(2) $$\begin{array}{l} f_{1}^{k}=\frac{d\left(1-\nu^{2}\right)}{E}\left[1+2\cos\left(2\theta_{k}\right)\right]\\ f_{2}^{k}=\frac{d\left(1-\nu^{2}\right)}{E}\left[1-2\cos\left(2\theta_{k}\right)\right]\\ f_{3}^{k}=\frac{4d\left(1-\nu^{2}\right)}{E}\sin\left(2\theta_{k}\right) \end{array}$$

where $U_{k}$ (*k* = 1, 2, 3) are the diametrical displacements of the borehole in the *k*th direction; *E* and *ν* are the Young’s modulus and the Poisson’s ratio of the rock, respectively; *d* is the diameter of the borehole; and $\theta_{k}$ (*k* = 1, 2, 3) are the three angles from the *x*-direction.

**Figure 2:**
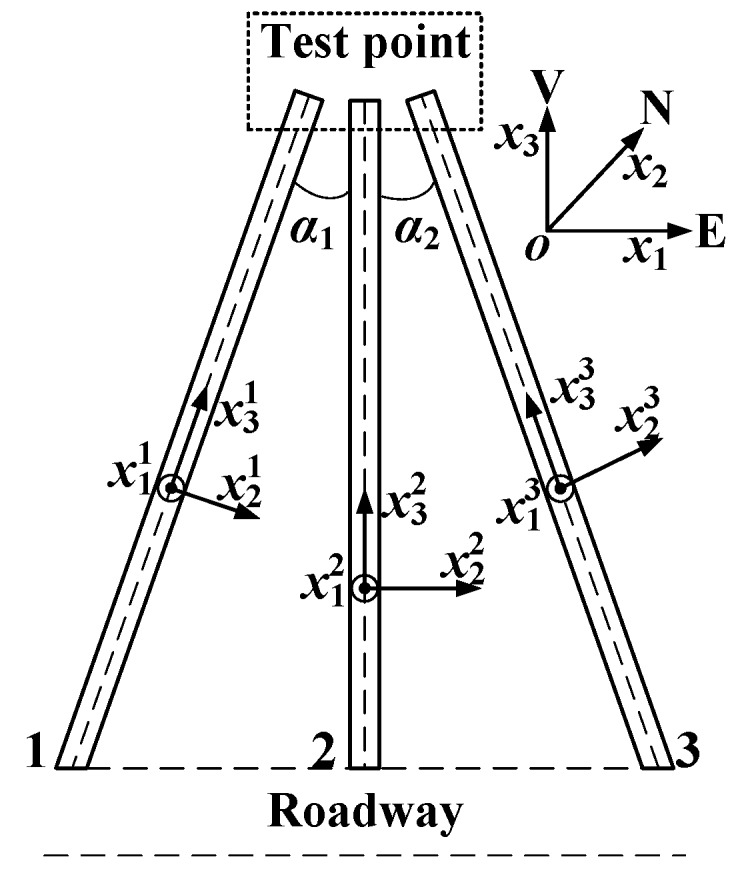
Three boreholes and local coordinate systems.

The stress tensor at a point within a rock mass is represented by a second-order Cartesian tensor with six independent components ($\sigma_{xx},\;\sigma_{yy},\;\sigma_{zz},\;\sigma_{xy},\;\sigma_{yz},\;\sigma_{xz}$) in the coordinate system shown in Figure 1 [27]. However, only three components ($\sigma_{xx},\;\sigma_{yy},\;\sigma_{xy}$) in the plane perpendicular to the borehole axis can be determined by Equations (1) and (2). Therefore, a minimum of three nonparallel boreholes are required to determine the complete stress tensor. As shown in Figure 2, the physical conditions present in three separate boreholes are assumed to prevail at one point in space. For field tests, the angles between three holes generally range from 20° to 60°. Three boreholes correspond to three local coordinate systems $x_{p}^{m}$ (*m* = 1, 2, 3; *p* = 1, 2, 3), and the east, north, up (ENU) coordinate is used as the global coordinate system, *x_i_* (*i* = 1, 2, 3). Each stress component in the local coordinate system can be expressed by the stress components in the global coordinate system according to the tensor transformation rule. The transformation rule can be written as:

(3) $$\sigma_{pq}^{m}=l_{pi}^{m}l_{qj}^{m}\sigma_{ij}$$

where *m* = 1, 2, 3 refers to the number of boreholes; the subscripts (*p* = 1, 2, 3; *q* = 1, 2, 3) indicate three coordinate axes in the local coordinate system; the subscripts (*i* = 1, 2, 3; *j* = 1, 2, 3) indicate three coordinate axes in the global coordinate system; $\sigma_{pq}^{m}$ is the stress tensor in the *m*th local coordinate system; $\sigma_{ij}$ is the stress tensor in the global coordinate system; and $l_{pi}^{m}$ is the cosine of the angle between unit vectors in the directions $x_{p}^{m}$ and $x_{i}$, respectively.

Substituting Equation (1) into Equation (3), the full stress tensor $\sigma_{ij}$ in a rock mass can be determined.

### 2.2. Measurement Methods of Stresses in Coal Mine Rock

Generally, stresses in rock can be divided into in situ stresses and induced stresses [2]. In situ stresses are the stresses that exist in the rock prior to any sort of disturbance. They are mainly due to the gravity and tectonic forces that result from the motion of crustal plates. Induced stresses are associated with artificial disturbances (excavation, drilling, mining, loading, etc.). Therefore, the real stresses in rock are:

(4) $$\sigma_{ij}^{r}=\sigma_{ij}+\Delta \sigma_{ij}\;\;\left(i,\;j=1,\;2,\;3\right)$$

where $\sigma_{ij}$ is the in situ stress tensor in rock and $\Delta \sigma_{ij}$ is the induced stress tensor in rock.

For stress in mine rock, the in situ stresses are almost kept constant, while the induced stresses can vary constantly owing to mining activities. Therefore, two steps should be conducted to obtain the real stress in mine rock. First, three sensors are installed in three nonparallel boreholes, and the in situ stresses are determined from borehole deformations created by the relief process. Subsequently, another three sensors are installed in the same boreholes to monitor the induced stresses created by mining activities.

## 3. FBG Borehole Deformation Sensor

### 3.1. Principle of the FBG Sensing Ring

The FBG is an optical filtering device that reflects light of a specific wavelength and is present within the core of an optical fiber waveguide. According to the Bragg condition, the shift in the Bragg grating center wavelength of the grating is

(5) $$\frac{\Delta \lambda_{B}}{\lambda_{B}}=\left(1-P_{e}\right)\varepsilon_{m}+\left(\alpha +\xi \right)\Delta T$$

where $\Delta \lambda_{B}$ is the shift in the wavelength of FBG; $\lambda_{B}$ is the central wavelength of FBG; $p_{e}$ is an effective strain-optic constant and $\varepsilon_{m}$ is the mechanical strain change; ΔT is the temperature change; α is the thermal expansion coefficient for the fiber; and ξ represents the thermo-optic coefficient.

**Figure 3:**
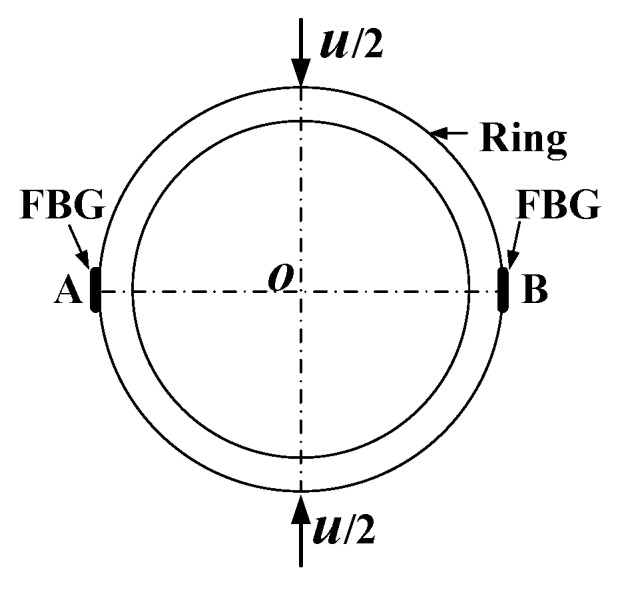
Drawing of principle of the fiber Bragg grating (FBG)-sensing ring.

As shown in Figure 3, when the diameter of the ring deforms (u), the maximum circumferential strain ($\varepsilon_{\theta }$) in the ring occurs at Points A and B where two FBGs are attached. The two FBGs at Points A and B can measure the circumferential strains in the ring. According to the elasticity theory and Equation (5), the wavelength shift of FBG at Point A or B ($\Delta \lambda_{B}$) varies linearly with the diametral displacement of the borehole:

(6) $$\Delta \lambda_{B}=K_{u}u$$

where $K_{u}$ is the deformation coefficient of the ring.

### 3.2. Sensor Design

**Figure 4:**
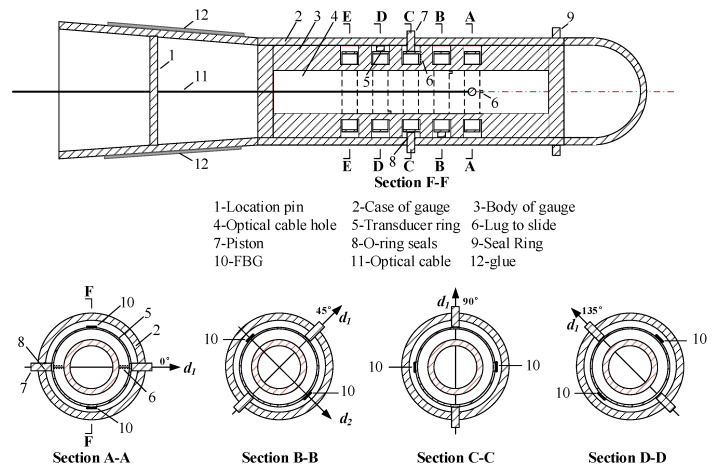
Drawing of principle parts of the FBG borehole deformation sensor.

**Figure 5:**
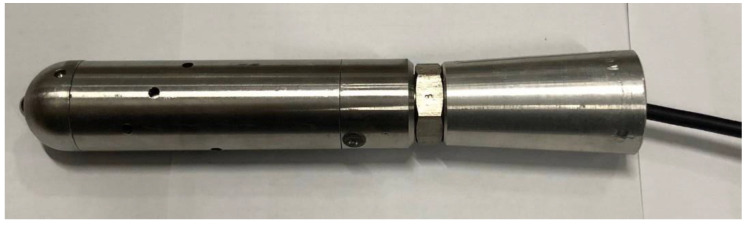
Photograph of the assembled FBG borehole deformation sensor.

The FBG sensor should be sensitive enough convert small borehole deformations into fiber strain. In addition, the sensor should be designed to reduce its creeping and moisture infiltration when used for long-term monitoring of stress changes. To achieve this goal, a new borehole deformation sensor based on FBG was developed. Figure 4 schematically illustrates the sensor scheme, and Figure 5 presents the assembled sensor, which fits into small pilot holes with a diameter of 36.0–37.0 mm. 

The case and the body of the sensor are made out of stainless steel and aluminum alloy, respectively. The measuring elements are four transducer rings that are made out of alloy spring steel. The diameter and thickness of the ring is 26.0 mm and 5.0 mm, respectively. The four rings can measure the diametral deformations of the borehole across four diameters spaced 45° apart in a plane perpendicular to the borehole axis. For each transducer ring, there are three FBGs inscribed in a single fiber. The fiber used for the sensor is an acrylate coated single-mode (SM) silica fiber. The grating is inscribed in the SM fiber, and its length is 8.0 mm. Two of them are bonded to the ring in a diametral direction (*d*_2_) using a type of epoxy glue, and they can measure the circumferential strain in the ring. Another one is a reference FBG subjected to the same thermal load but free from mechanical load. For each ring, two pistons press against the ring in a diametral direction (*d*_1_) that is perpendicular to the *d*_2_ direction. When installing the sensor, the location pin can be used to determine the orientation of the sensor in a pilot hole. The length of the piston is carefully selected to ensure that the length of the piston plus the diameter of the ring is larger than the diameter of the borehole. After the sensor is installed in a borehole, the ring can produce a contact force for two pistons, hence ensuring perfect contact between the piston and the rock. When the stresses change in the rock, the diameter of the borehole changes accordingly. The pistons move, and hence different strains are induced in the bonded FBGs. From the wavelength shifts of FBGs, and with the use of elasticity theory, the stresses in the plane perpendicular to the borehole axis can be determined.

Provisions have been taken to protect the sensor from corrosion, water and dust. The glue at the end of the sensor, the O-ring seals and the seal ring at the front of the sensor can all help in resisting corrosion and vibration for the long-term monitoring of stress changes. 

### 3.3. Two Built-in Checks on Test Data

For the developed sensor, two very simple checks on the accuracy of the test are done.

(1) Owing to the lug to slide (6 in Figure 4), the transducer ring can only move in the *d*_1_ direction. Moreover, two bonded FBGs are symmetrical with respect to the *d*_1_ direction, and their attachments to the ring are nearly consistent. As a result, the wavelength shifts of two bonded FBGs vary equally when the borehole deforms:

(7) $$\Delta \lambda_{i}^{1}=\Delta \lambda_{i}^{2}\;\;\left(i=1,\;2,\;3,\;4\right)$$

where *i* = 1, 2, 3, 4 refers to four transducer rings and $\Delta \lambda_{i}^{1}$ and $\Delta \lambda_{i}^{2}$ are the wavelength shifts of two bonded FBGs, respectively.

(2) From Equation (1), the sum of any two diametral deformations spaced 90° apart in the pilot hole must be constant. In other words, the sum of the diametral displacements recorded by the first and the third rings are equal to the sum recorded by the other two rings.

(8) $$U_{\theta =0{}^{\circ}}+U_{\theta =90{}^{\circ}}=U_{\theta =45{}^{\circ}}+U_{\theta =135{}^{\circ}}$$

For any particular tests, Equations (7) and (8) can be used to check the test data. Only when both equations have been satisfied within the limits of experimental error, can the test data be considered reliable. Then, any three of the four measured diametral displacements can be selected to determine the rock stresses by Equations (1) and (3). The feature of two built-in checks can bring more reliable and accurate test result, and it represents an improvement over the existing sensors.

### 3.4. Accuracy Calibration

**Figure 6:**
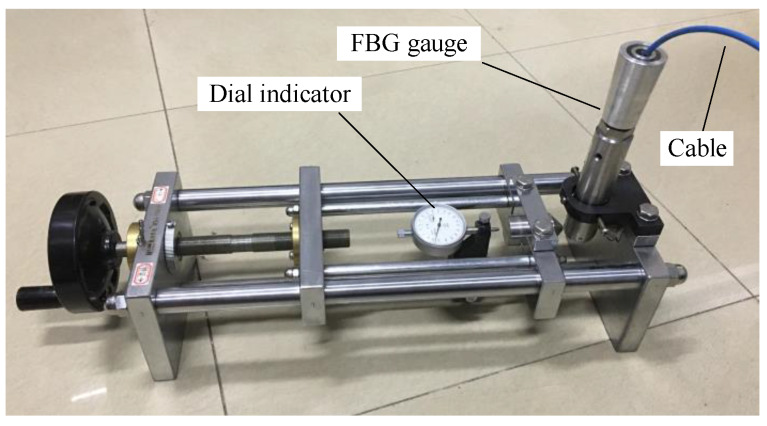
Calibration setup.

The strain sensitivity of the FBG borehole deformation sensor is significantly different from that of the bare FBG. Meanwhile, the strain sensitivity of the sensor is affected by the geometric characteristics of transducer rings, the location of FBG, and the attachment between the ring and FBG. Hence calibration of the sensor must be carried out before it is put into use, though the difference between sensors is small. The calibration procedure is as follows:

(1) As shown in Figure 6, the sensor was placed into the calibration setup, which can apply different displacement on the pistons, and was wired to the interrogation system. The interrogation system used in this study was based on a Fabry–Perot laser light source and had a resolution of 1.0 pm. An initial displacement was applied onto the two pistons, and the wavelengths of FBGs for the initial conditions were recorded.
(2) A certain amount of displacement is applied onto the pistons in the *d*_1_ direction (Figure 4), step by step, until the predesigned displacement was reached (0.1 mm for each step and a total of 1.0 mm). For each step, the displacement was maintained for 5 min, and the wavelengths of FBGs were recorded twice. The first was recorded immediately after the displacement was applied, and the second was recorded after 5 min. The stability of the wavelengths was checked.
(3) Backing out of the applied displacement step by step (0.1 mm for each step). Similarly, for each step, the wavelengths of FBGs were recorded twice, and the stability of wavelengths were checked as well. This procedure was continued with the same increments until the initial point on the micrometer was reached. This zero displacement will be the zero-displacement reading for the second run.
(4) The relationship between the applied displacement and the wavelength shift of FBG can be obtained.
(5) The above calibration procedure is then repeated for the other transducer rings. Since each ring of the sensor is calibrated separately, the predefined spacing between transducer rings inside the sensor has no effect on the accuracy of the sensor.

(1) Accuracy and range

**Figure 7:**
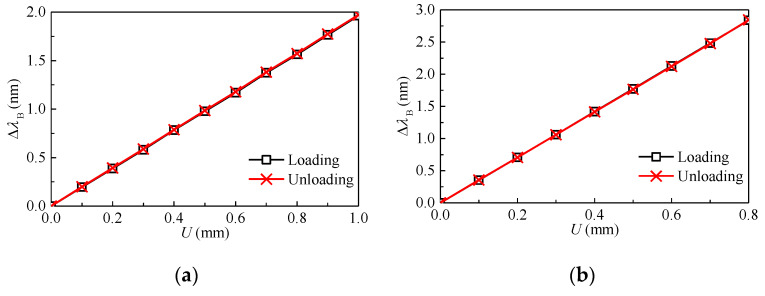
Relationship between the diametral displacement of ring and the wavelength shift of FBG. (**a**) Ring with a thickness of 0.6 mm; (**b**) ring with a thickness of 1.0 mm.

Typical calibration curves can be found in Figure 7. The wavelength shift of FBG with the applied diametral displacement is found to be perfectly linear, and the relationship can be written as:

(9) $$\Delta \lambda_{i}=K_{u}^{i}U_{i}\;\left(i=1,\;2,\;3,\;4\right)$$

where $K_{u}^{i}$ is the deformation coefficient of the *i*th transducer ring.

As stated above, the sensor should have a sufficient accuracy to determine the stresses in rock. In addition, the range of the sensor should be wide enough as well. It can be seen from Figure 7 that for a ring with a thickness of 0.6 mm, the wavelength shift corresponds to approximately 2.0 nm for a displacement change of 1.0 mm. For the interrogation system with a resolution of 1 pm, the sensor accuracy is 0.5 × 10^−3^ mm. This accuracy is sufficient enough to obtain an estimate of the stress in the rock. For practical applications, the contact force between the piston and the rock is very small (smaller than 100 N). Therefore, the contact force has no effect on the rock deformation, and the diametral deformation of the ring is equal to the rock deformation. For a given diametral deformation, as the thickness of the ring increases, the circumference strain of the ring and the wavelength shift of FBG increase. As a result, the sensor becomes more sensitive to the deformation. As indicated in Figure 7, the accuracy for a ring with a thickness of 1.0 mm is approximately 0.3 × 10^−3^ mm.

### 3.5. Temperature Compensation

**Figure 8:**
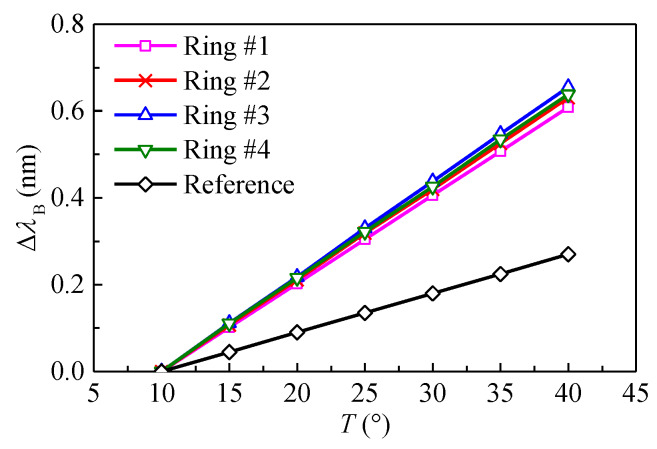
Variation of wavelength shifts of FBGs with temperature.

Since the FBG is sensitive to strain as well as temperature, a practical method should be used to compensate the temperature effect. A simple solution is to introduce a reference FBG that is identical to the strain-measuring FBG but free from mechanical strain [28]. As shown in Figure 4, for the FBG borehole deformation sensor in this study, a reference FBG is introduced on each ring to separate the two contributions. A laboratory test was conducted to investigate the temperature sensitivity of different FBGs. The sensor was placed in a thermostat, while the interrogation system was placed outside the thermostat where the temperature was nearly unchangeable. The thermostat was set to different temperatures and the wavelength shifts of the FBGs were recorded under different temperatures. The relationship between the wavelength shifts of the FBGs and the temperature can be found in Figure 8. As the temperature increases, the wavelengths of different FBGs increase linearly. The relationship can be written as:

(10) $$\begin{array}{l} \Delta \lambda_{i}=K_{T}^{i}\Delta T\;\left(i=1,\;2,\;3,\;4\right)\\ \Delta \lambda_{i,R}=K_{T}^{i,R}\Delta T\;\left(i=1,\;2,\;3,\;4\right) \end{array}$$

where $\Delta \lambda_{i}$ and $\Delta \lambda_{i,R}$ are the wavelength shifts of the bonded and reference FBGs corresponding to the *i*th ring, respectively; and $K_{T}^{i}$ and $K_{T}^{i,R}$ are the temperature coefficients of the bonded and reference FBGs corresponding to the *i*th ring, respectively.

It can be seen from Figure 8 that compared with the reference FBG, the bonded FBGs are more sensitive to temperature. The temperature coefficients of bonded FBGs are nearly equivalent to each other. From Equations (9) and (10), the diametral displacement of the transducer ring induced by only the mechanical load can be written as:

(11) $$U_{i}=\frac{1}{K_{u}^{i}}\left[\Delta \lambda_{i}-\frac{K_{T}^{i}}{K_{T}^{i,R}}\Delta \lambda_{i,R}\right]\;\left(i=1,\;2,\;3,\;4\right)$$

## 4. Laboratory Tests on the FBG Sensor

A laboratory test was conducted to verify the performance of the presented sensor when used for measuring the stress in a borehole. 

### 4.1. Description of the Model Test

**Figure 9:**
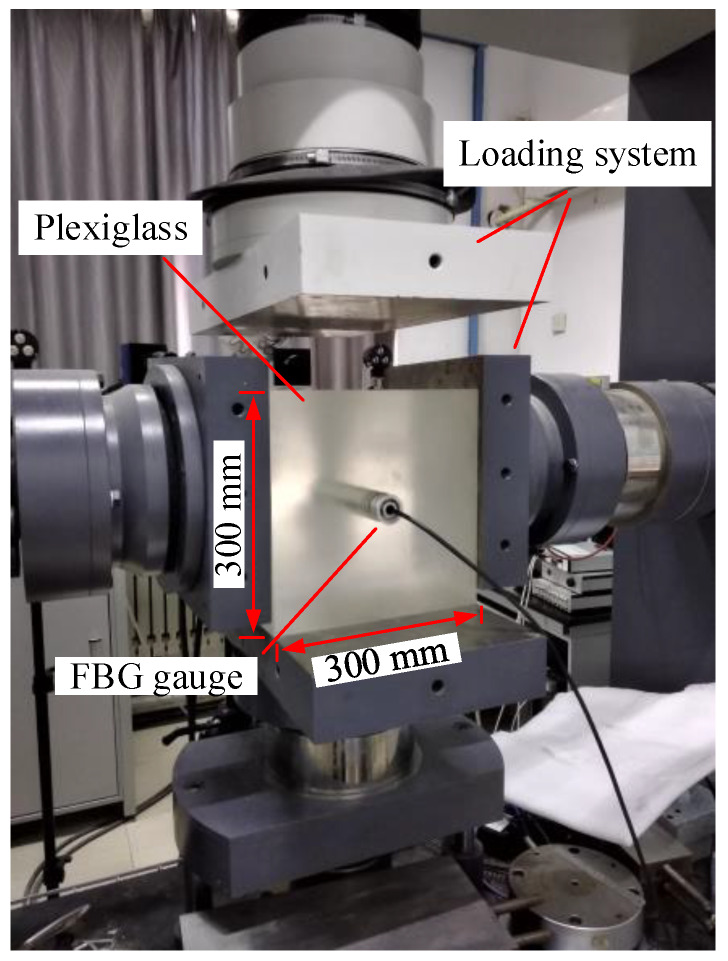
Experiment setup and plexiglass block containing a FBG sensor.

The model test was conducted using a stiff servo-controlled testing machine at Institute of Rock and Soil Mechanics, Chinese Academy of Sciences (Figure 9). The machine can perform biaxial compression tests on rock, concrete and other materials. Two loading systems were used to apply the forces in the horizontal and vertical directions, respectively. The machine provides force capacity of 1000 kN in two directions. The process of the model test was as follows:

(1) Since the plexiglass is homogeneous and isotropic, as shown in Figure 9, a Plexiglass block (300 mm × 300 mm × 250 mm) was prepared. The Plexiglass has a Young’s modulus of 3.1 GPa and a Poisson’s ratio of 0.23.
(2) A small hole with a diameter of 36.5 mm was drilled at the center of one face. The sensor was then placed into the small hole and wired to the interrogation system.
(3) The Plexiglass block containing the sensor was placed on the stiff servo-controlled testing machine. For the tests in this study, only the vertical load (*p*) was applied by the vertical actuator, and the wavelengths of the FBGs were monitored.
(4) The stresses in the plane perpendicular to the hole axis were calculated by the wavelength shifts of FBGs, and the normal stress (*σ_xx_*) in the vertical direction was compared with the applied load (*p*).

### 4.2. Test Results

**Figure 10:**
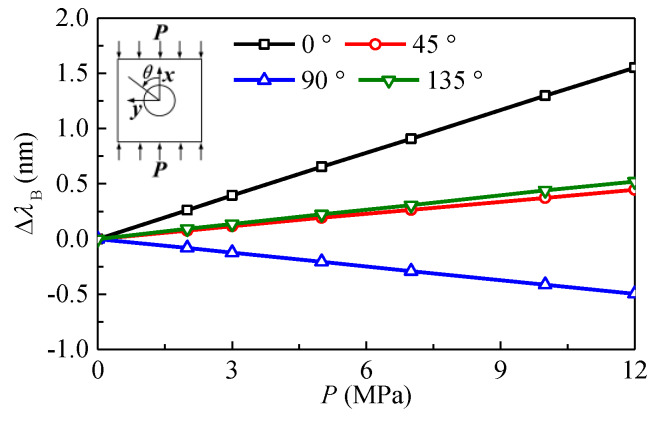
Variation of wavelength shifts of FBGs bonded onto four rings with applied loads.

The test results are shown in Figure 10. The thickness of the transducer ring is 1.0 mm for this experiment. It can be seen that the wavelength shifts of FBGs depend linearly on the applied load. The increase in the wavelength of FBG indicates the decrease in the diameter of the hole. Therefore, the wavelength of FBG at 0° increases but decreases at 90°, which indicates that the diameter of the hole at 0° decreases but increases at 90°. In addition, the diametral displacement at 45° is nearly equal to that at 135°.

**Figure 11:**
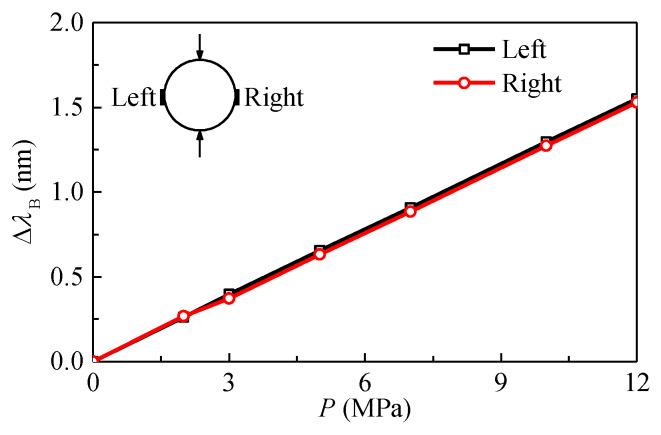
Variation of wavelength shifts of two FBGs bonded on the same ring with applied loads.

**Figure 12:**
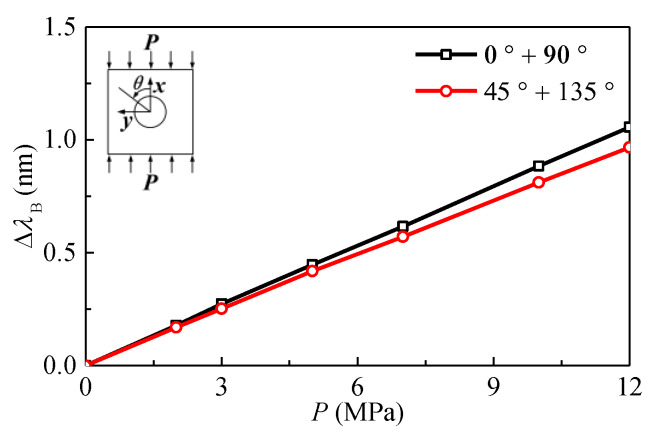
Variation of wavelength shifts of FBGs spaced 90° apart with applied loads.

The test data was checked using Equations (7) and (8) before calculating the stresses. It can be seen from Figure 11 that the wavelength shifts of the two FBGs bonded onto the same ring are nearly equal to each other. In addition, as shown in Figure 12, the wavelength shifts of FBGs bonded onto four different rings satisfy the conditions in Equation (8) within the limits of test error. The results indicate that the test data is reliable and can be used to calculate the stresses in the plexiglass.

The calculated stresses for different applied loads are shown in Table 1. It can be seen that the normal stress in the *x* direction (*σ_xx_*) is close to the applied load, and the other two stress components are much smaller. The maximum deviation with respect to the applied load is approximately 6%, which indicates that the sensor has a good accuracy when used for measuring stress components in the plane perpendicular to the borehole axis.

## 5. Application in a Coal Mine

### 5.1. Description of the Coal Mine 

The Dongtan Mine that is an underground coal mine is located in Ji-ning, China. The 6305 mining face was selected to check the field performance of the developed sensor and investigate the variation of stress in the roof owing to mining activities. The size of the mining face is approximately 250 m × 1500 m, and the buried depth of the coal seam is approximately 660 m. The average thickness of the coal seam is approximately 5.0 m. The major lithological units in the roof are siltstone and sandstone rocks of fair to good quality rock masses.

### 5.2. Test Procedure

**Figure 13:**
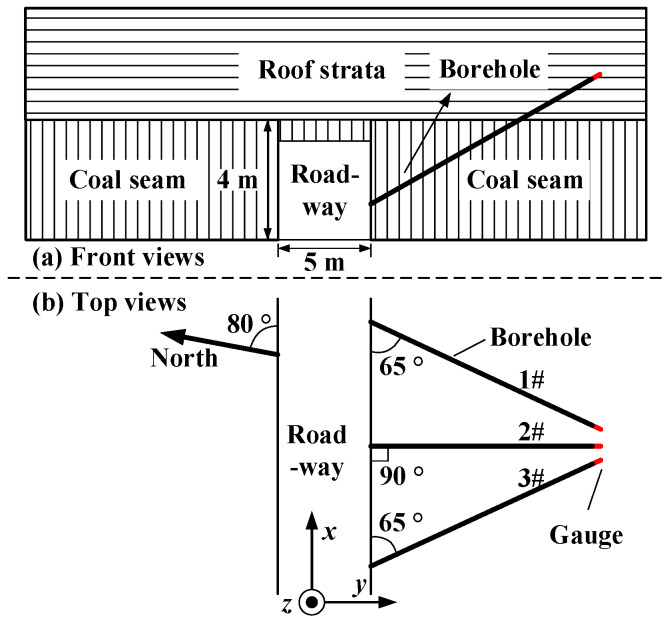
The layout of three boreholes and the local coordinate system.

The locations and orientations of the three nonparallel boreholes are shown in Figure 13. The dip angles of the three boreholes are 30°, and the angle that the middle borehole makes with the other two is 25°. The three pilot holes are located in a small area in space. The real stresses in the roof are the sum of the in situ stresses and the induced stresses. Therefore, two steps were taken to obtain the real stresses in the roof. First, three borehole deformation sensors were installed in the three boreholes, and the in situ stresses were determined from the borehole deformations created by the relief process. Subsequently, another three sensors were installed into the same three boreholes, and the induced stresses were monitored from the borehole deformations created by artificial mining over a long period of time. For the long-term monitoring of the induced stresses, the sensors were connected to an intrinsically safe interrogator unit through an optical cable. The interrogator unit was placed in an underground substation where the power supply unit was intrinsically safe.

### 5.3. Stresses in Rock

**Figure 14:**
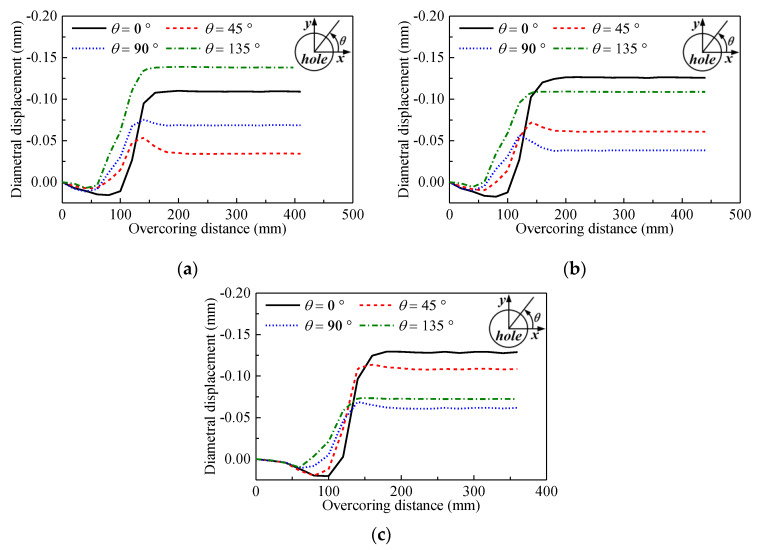
Overcoring test curves. (**a**) #1 borehole; (**b**) #2 borehole; (**c**) #3 borehole.

Since the rock mass was intact and homogeneous in the test site, the overcoring tests in the three boreholes were successful. The recorded wavelength shifts of FBGs were converted into the diametral displacements in different directions using Equation (11), and the test curves after the temperature compensation for the three boreholes are shown in Figure 14. It can be seen that all curves present regular shapes. Positive (compressive) displacement occurs before the overcoring bit passed the measuring plane. When the overcoring bit approaches the measuring plane, the diametral displacement changes to become negative (tensile) and increases rapidly to the peak point. After approximately 200 mm of overcoring, the diametral displacement does not change much with further drilling.

The calculated diametral displacements were first checked according to Equations (7) and (8) before being used to calculate the stresses. It can be seen from Table 2, Table 3 and Table 4 that the two diametral displacements determined by the two FBGs (No.1 left and No.2 right) bonded onto the same ring are nearly equal to each other. In addition, the diametral displacements in the last two columns (Table 2, Table 3 and Table 4) are nearly equal as well. The test data satisfies two conditions in Equations (7) and (8) within the limits of test error. In addition, the test data in any three directions with large wavelength shifts of FBGs should be selected to reduce the effect of random error on the test results. Therefore, the diametral displacements at 0°, 90° and 135° of the first borehole (Table 2) and the diametral displacements at 0°, 45° and 135° of the other two boreholes (Table 3 and Table 4) were selected to calculate the in situ stress components ($\sigma_{xx}^{m},\sigma_{yy}^{m},\tau_{xy}^{m}$; *m* = 1, 2, 3) in the planes perpendicular to three borehole axes. The stress components in the local coordinate shown in Figure 13 were calculated according to the tensor transformation rule in Equation (3). The stress components in the local coordinate system shown in Figure 13 are presented in Table 5. 

After the overcoring test, another three sensors were installed into the same three boreholes approximately 250 m ahead of the mining face to monitor the induced stresses, $\Delta \sigma_{ij}$ (*i, j = x, y, z*), in the roof. From Equation (4), the real stresses, $\sigma_{ij}^{r}$ were obtained by adding the in situ stress components, $\sigma_{ij},$ in Table 5 and the induced stress components, $\Delta \sigma_{ij}$. For the long-term monitoring of stress changes in mine rock, the sensors were installed in roof, and they were connected to an intrinsically safe interrogator unit through an optical cable. The interrogator unit was placed in an underground substation where the power supply unit was intrinsically safe. All the wavelengths of FBGs obtained by the interrogator unit were transmitted to a computer in the aboveground control room through the local area network, and the data was processed and displayed by a computer in the control room.

**Figure 15:**
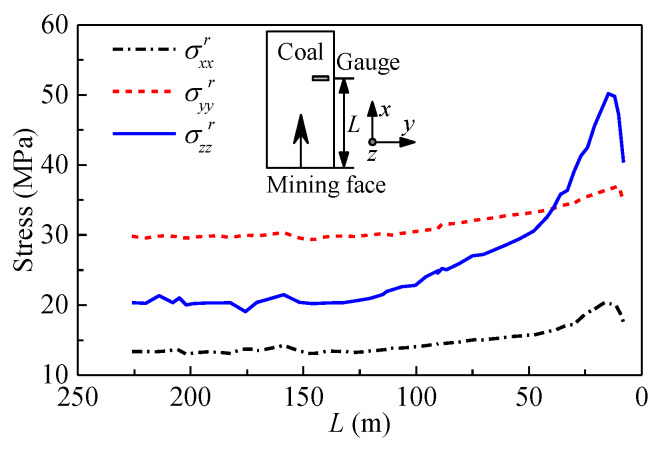
Variations of three normal stresses in the roof with distance from the mining face.

The long-term changes of the three normal stresses ($\sigma_{xx}^{r},\;\sigma_{yy}^{r},\;\sigma_{zz}^{r}$) in the local coordinate system are shown in Figure 15. When the distance from the mining face (*L*) is longer than 150 m, the three normal stresses are almost equivalent to the corresponding components of the in situ stresses. As the mining face further approaches the sensors, the rock pressure applied onto the coal seam near the mining face moves ahead. Consequently, all three normal stresses gradually increase. Moreover, the normal stress in the vertical direction increases much more rapidly than the other two stresses. When the distance ranges between approximately 50 m to 15 m, the three normal stresses increase rapidly with the decrease of the distance. In addition, the normal stress in the vertical direction exceeds the normal stress in the *y*-direction. Once the distance reaches approximately 15 m, the rock and coal near the sensors begin to break down, and three normal stresses start decreasing sharply owing to the rock fracture. For the peak point, three normal stresses increase by approximately 50%, 25%, and 150%, respectively. The variation of stresses in the roof was better understood with the aid of the developed FBG sensor, and it may assist in the design of the roadway supporting and the prevention of rock bursts. 

## 6. Conclusions

A borehole deformation sensor for long-term stress monitoring based on FBG was presented in this study. The main conclusions can be summarized as follows:

(1) The sensor converts borehole deformations into optical fiber strain by means of four transducer rings. For each ring, two FBGs are bonded with the ring to measure the strain in the transducer ring, and a reference FBG subjected to the same thermal load but free from mechanical load is introduced to remove the influence of temperature. The wavelength shift of the bonded FBG with applied diametral displacement or temperature is perfectly linear, and the sensor has an accuracy of approximately 0.3–0.5 × 10^−3^ mm, which corresponds to a sufficient stress accuracy for a mine rock.
(2) The sensor contains two built-in checks on the accuracy of the test. First, the wavelength shifts of two bonded FBGs on each ring vary equally when the borehole deforms. Second, the sum of the diametral displacements recorded by the first and the third rings are equal to the sum recorded by the other two rings. The two checks on the tests data are easily to perform, and they can ensure the reliability and accuracy of test results.
(3) The developed sensor can withstand downhole conditions in coal mines, and it performed well in the field over the whole mining process. By using the presented sensor, the in situ stresses in the roof of a coal mine were determined, and the long-term stress changes in the roof were also monitored. These results may help in the design of the roadway supporting and the prevention of rock bursts.

## Figures and Tables

**Table 1:** Comparison between applied load and calculated stress.

Load, *p* MPa	*σ_xx_* MPa	*σ_yy_* MPa	*τ_xy_* MPa	Deviation %
2.0	2.11	−0.01	−0.03	5.33
3.0	3.18	−0.05	−0.03	6.12
5.0	5.26	−0.08	−0.05	5.23
7.0	7.26	−0.19	−0.08	3.74
10.0	10.38	−0.31	−0.13	3.75
12.0	12.40	−0.40	−0.13	3.31

**Table 2:** Diametral displacements at different angles for the first borehole (Unit: mm).

	0°	45°	90°	135°	0° + 90°	45° + 135°
No.1 (left)	−0.108	−0.029	−0.070	−0.138	−0.178	−0.167
No.2 (right)	−0.110	−0.039	−0.066	−0.138	−0.176	−0.177
Average	−0.109	−0.034	−0.068	−0.138	−0.177	−0.172

**Table 3:** Diametral displacements at different angles for the second borehole (Unit: mm).

	0°	45°	90°	135°	0° + 90°	45° + 135°
No.1 (left)	−0.128	−0.063	−0.039	−0.109	−0.170	−0.172
No.2 (right)	−0.124	−0.061	−0.031	−0.107	−0.158	−0.168
Average	−0.126	−0.062	−0.035	−0.108	−0.161	−0.170

**Table 4:** Diametral displacements at different angles for the third borehole (Unit: mm).

	0°	45°	90°	135°	0° + 90°	45° + 135°
No.1 (left)	−0.126	−0.107	−0.065	−0.068	−0.191	−0.175
No.2 (right)	−0.130	−0.109	−0.057	−0.078	−0.187	−0.187
Average	−0.128	−0.108	−0.061	−0.073	−0.189	−0.181

**Table 5:** Stress components in the local coordinate system.

Stress Components	*σ_xx_*	*σ_yy_*	*σ_zz_*	*τ_xy_*	*τ_yz_*	*τ_xz_*
Magnitude/MPa	13.82	29.35	20.32	−2.73	0.03	2.77
